# Characterization of SARS-CoV-2 replication complex elongation and proofreading activity

**DOI:** 10.1038/s41598-022-13380-1

**Published:** 2022-06-10

**Authors:** Alisha N. Jones, André Mourão, Anna Czarna, Alex Matsuda, Roberto Fino, Krzysztof Pyrc, Michael Sattler, Grzegorz M. Popowicz

**Affiliations:** 1 Institute of Structural Biology, Helmholtz Zentrum München, Ingolstädter Landstr. 1, 85764 Neuherberg, Germany; 2 Department of Chemistry, Bavarian NMR Center, Technical University of Munich, Lichtenbergstraße 4, 85747 Garching, Germany; 3 Virogenetics Laboratory of Virology, Malopolska Centre of Biotechnology, Jagiellonian University, Gronostajowa 7a, 30-387 Kraków, Poland

**Keywords:** Viral infection, Proteins, RNA

## Abstract

The replication complex (RC) of SARS-CoV-2 was recently shown to be one of the fastest RNA-dependent RNA polymerases of any known coronavirus. With this rapid elongation, the RC is more prone to incorporate mismatches during elongation, resulting in a highly variable genomic sequence. Such mutations render the design of viral protein targets difficult, as drugs optimized for a given viral protein sequence can quickly become inefficient as the genomic sequence evolves. Here, we use biochemical experiments to characterize features of RNA template recognition and elongation fidelity of the SARS-CoV-2 RdRp, and the role of the exonuclease, nsp14. Our study highlights the 2′OH group of the RNA ribose as a critical component for RdRp template recognition and elongation. We show that RdRp fidelity is reduced in the presence of the 3′ deoxy-terminator nucleotide 3′dATP, which promotes the incorporation of mismatched nucleotides (leading to U:C, U:G, U:U, C:U, and A:C base pairs). We find that the nsp10–nsp14 heterodimer is unable to degrade RNA products lacking free 2′OH or 3′OH ribose groups. Our results suggest the potential use of 3′ deoxy-terminator nucleotides in RNA-derived oligonucleotide inhibitors as antivirals against SARS-CoV-2.

## Introduction

Coronaviruses are amongst the largest known RNA viruses, with a genome size reaching 30,000 nucleotides (nt)^1^. They replicate solely in the cytoplasm, where the viral RNA is multiplicated, proteins are produced, matured, and assembled to form new viral particles^2,3^. Replication of the viral genome starts with the generation of the negative (-) RNA strand, starting from the 3′ end^4^. During this process, the replication complex (RC) produces full-length genomes but due to employment of the discontinuous transcription mechanism, a set of shorter sub-genomic (sg) mRNAs is also formed. In a proportion of cases, the RC stops at the transcription-regulating sequence (TRS) elements and switches the template, adding the leader fragment from the 5′ end of the genome to a truncated mRNA molecule. TRS sequences are located before each functional open reading frame. Consequently, a subset of mono-cistronic sg mRNAs is produced, and the sequence of the TRS tightly regulates the quantity of each transcript. Resulting RNAs are transcribed again, to yield the plus (+) stranded RNA that serves as a template for protein production. This discontinuous transcription process is very peculiar and not fully understood, but it also constitutes an elegant and compact system allowing for an efficient regulation of protein production.

Recent structural studies have revealed important features of RNA binding by the SARS-CoV-2 RNA-dependent RNA polymerase (RdRp), comprised of the nsp12 polymerase and the nsp7 and two nsp8 cofactors^5–8^. The structure reveals that the nsp12 polymerase forms a tight complex with nsp7 and nsp8. While the function of nsp7 is not clear, the two copies of nsp8 form extended helices that serve as RNA-binding interfaces; the extended helices may also serve as sliding poles that stabilize the RNA template/product duplex emerging from the nsp12 catalytic core^6,7^. This unique feature has been proposed to ensure and enhance processivity for replication of the large SARS-CoV-2 genome. Nps8 was also postulated to possess polymerase/primase activity based on hexadodecameric self-assembly^9,10^. This implies that nsp7–nsp8 might have two functions that are structurally completely independent.

The SARS-CoV-2 RC was recently shown to be the fastest RdRp of any known coronavirus, elongating RNA products at 600 nts/sec^11,12^. The RdRp has also been reported to exhibit low nucleotide insertion fidelity. Low replication fidelity has been proposed to allow RNA viruses to quickly adapt to different (host) environments and selection pressures. This requires a fine balance between genome diversity and fitness for virulence and evolution^13,14^. As a consequence of the poor replication fidelity, the SARS-CoV-2 RC tolerates the incorporation of RdRp inhibitor nucleoside analogs including Favipiravir and Remdesivir, which confer C-to-U and G-to-A transitions^8,11,15^. In response to the low replication fidelity, there exists an exonuclease, nsp14, which is conserved in coronaviruses, and functions to proofread and exclude mismatched nucleotides during the replication process^14,16^. Nsp14 is peculiar in that it combines two enzymatic properties: 3′–5′ exonuclease activity and methyltransferase activity, which is responsible for adding a 5′ cap to the nascently transcribed viral RNA to enable its translation by the host machinery. The nsp14 exonuclease activity is strongly enhanced by a protein–protein interaction with the nsp10 cofactor, and is proposed to interact with the RdRp^13,17–21^.

Here, we investigate the molecular features required for RNA recognition and replication fidelity of the SARS-CoV-2 RdRp with naturally-occurring and ribose-modified substrates and oligo-nucleotides. Furthermore, we characterize the exonuclease activity of nsp10–nsp14 proofreading function in the presence of the polymerase. Our results show that the RdRp binds with micromolar affinity to its RNA substrates and that the 2′OH group of the ribose backbone is critical for elongation activity. Chemically stabilized RNA analogues are preferentially bound to nsp12. We find that the RdRp indiscriminately incorporates the adenosine derivative Cordycepin (3′-deoxyadenosine 5′-triphosphate), which results in the premature termination of an elongated RNA product and promotes U:U, U:G, and U:C mismatches. Interestingly, the nsp10–nsp14 exonuclease heterodimer is unable to degrade RNAs possessing terminal 3′ deoxynucleotides. This suggests that these nucleosides could serve as a SARS-CoV-2 antiviral agent through interference with RdRp machinery. The exonuclease activity depends on the RNA sequence and the recognition of free 2′OH and 3′OH ribose groups. Altogether, our results provide insight into the molecular mechanisms and chemical specificity of the SARS-CoV-2 RdRp and the nsp10–nsp14 exonuclease complexes. This opens a possibility to design specific RdRp inhibitors based on oligonucleotide analogues as novel antiviral drugs.

## Results

### The SARS-CoV-2 RdRp binds RNA substrates with low micromolar binding affinity and relies on recognition of the 2′OH of the ribose

**Figure 1:**
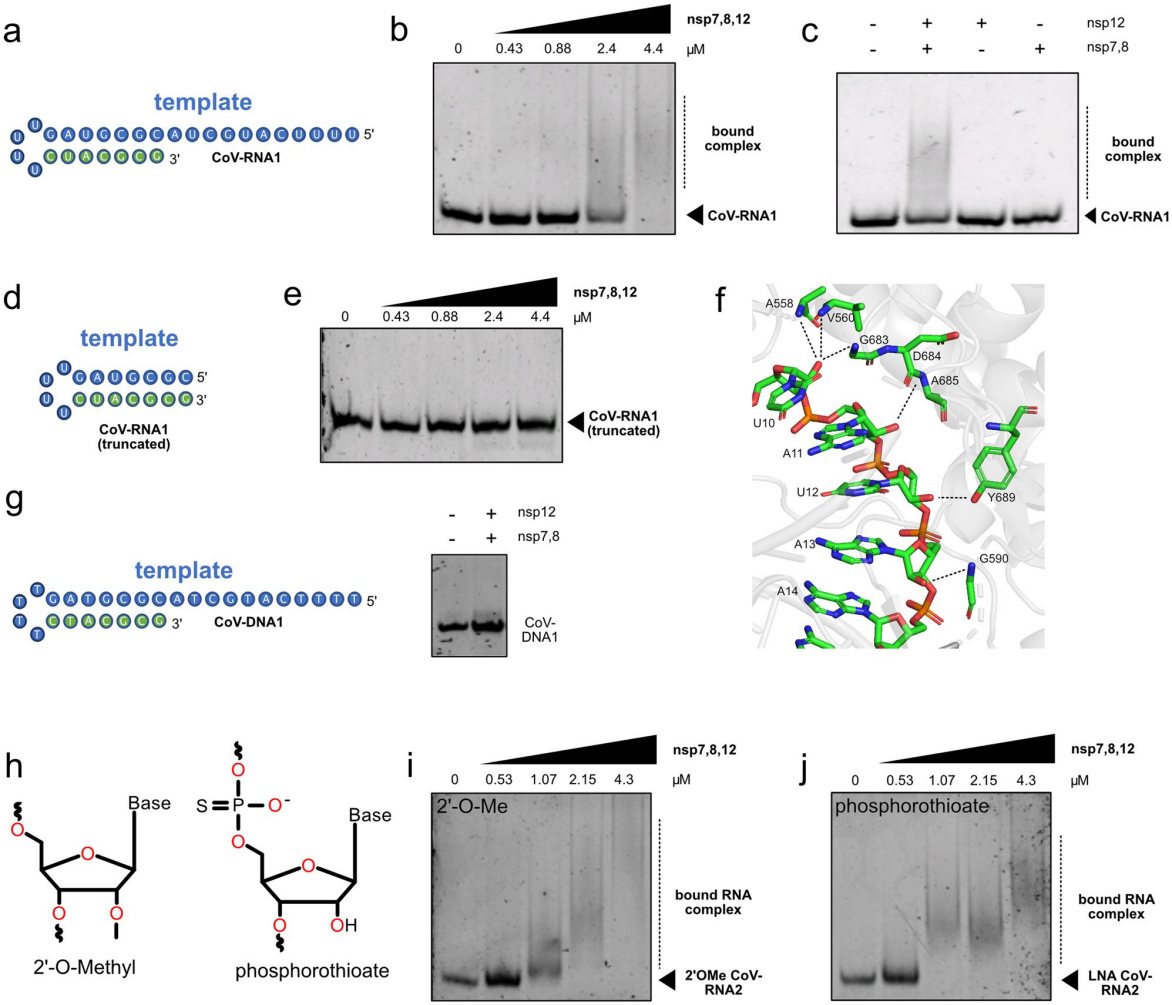
Nsp7,8,12 binds RNA substrates possessing a 5′ single-stranded overhang. (**a**) The secondary structure of a minimal RNA substrate recognized by the SARS-CoV-2 RdRp, coined CoV-RNA1. (**b**) Nsp7,8,12 binds the CoV-RNA1 substrate between 2.4 and 4.4 μM. (**c**) CoV-RNA1 binds nsp7,8,12 (2.4 µM) but not nsp12 alone (2.4 µM) or to the nsp7,8 protein–protein complex (2.4 µM). (**d**) The secondary struture of a trunctated CoV-RNA1 construct, lacking the 5′ single-stranded overhang. (**e**) Nsp7,8,12 does not bind the truncated CoV-RNA1 substrate. (**f**) Nsp12 forms contacts (i.e. H-bonds and electrostatic interactions) with RNA through the 2′OH group of the ribose (PDB 7BV2^8^). (**g**) The secondary structure of a DNA substrate, coined CoV-DNA1, is not recognized by nsp7,8,12 (2.4 µM). (**h**) The ribose modifications, 2′-OMe or phosphorothioate, that are incoporated into the CoV-RNA2 substrate. (**i**, **j**) 2′O-Me and phosphorothioate CoV-RNA2 substrates bind to nsp7,8,12 as low as 1.07 μM.

Based on a previously described SARS-CoV-2 RNA substrate^7^ (herein referred to as CoV-RNA1, Fig. 1a), we designed various variant RNA and DNA substrates to evaluate the binding activity of the SARS-CoV-2 RdRp (Table 1). In electrophoretic mobility shift assays (EMSA) we observed a moderately weak interaction between the nsp7,8,12 complex and CoV-RNA1, where broadening of shifted bands corresponding to fast-exchanging complexes were observed between 2.4 and 4.4 μM (Fig. 1b). A modest binding affinity is not surprising as it likely allows for a rapid rate of RdRp elongation^11^. No RNA binding was detected for nsp12 alone or nsp7,8 with the CoV-RNA1 substrate (Fig. 1c). An EMSA run with the RdRp and the CoV-RNA1 substrate lacking a 5′ overhang showed no retardation of a complex (Fig. 1d, e). Further evaluation of stem-loop structures with blunt ends that are recognized by structurally similar RdRps (i.e. the hepatitis C viral polymerase ns5b) also showed no significant binding activity (Supplementary Fig. 1a), despite the two polymerases being highly similar^22^.

**Table 1:** RNA and DNA substrates used in binding and elongation assays.

Substrate	Primary sequence (5′–3′)
COV-RNA1	UUUUCAUGCUACGCGUAGUUUUCUACGCG
COV-DNA1	TTTTCATGCTACGCGTAGTTTTCTACGCG
COV-RNA1—TRUNCATED	CGCGUAGUUUUCUACGCG
COV-RNA1-X (X = G, U, A, or C)	XXXXXXXXXXXCGCGUAGUUUUCUACGCG
COV-RNA1-TERM	UUUUCAUGCUACGCGUAGUUUUCUACGCG(3′drU)
COV-RNA2	UUUUCUACGCGUAGUUUUCUACGCG
2′-OME COV-RNA2	UUUUCUACGCGUAGUUUUCUACGCG
PHOSPHOROTHIOATE COV-RNA2	UUUUCUACGCGUAGUUUUCUACGCG

We next investigated whether the identity of the 2′ substituent of the ribose influences recognition by the RdRp, as it has been reported that several residues of nsp12 recognize the 2′ OH group of RNA (Fig. 1f)^6–8^. To this end, we evaluated binding with a variant DNA substrate (CoV-DNA1) and RNA substrates possessing modified riboses (Table 1). No binding was observed between CoV-DNA1 and the SARS-CoV-2 RdRp (Fig. 1g), supporting the notion that the 2′OH of the ribose is essential for recognition by the RdRp. EMSAs with an RNA substrate possessing 2′-O-methyl groups (2′-OMe CoV-RNA2) at the C2′ positions of the riboses retains binding with micromolar affinity to RdRp, with a poorly associated complex formed at an RdRp concentration as low as 1 μM, with no free RNA remaining by 2.15 μM (Fig. 1h, i). Furthermore, replacing the riboses with phosphorothioate nucleic acid variants in CoV-RNA2 (an RNA substrate with a 5′ overhang four nucleotides shorter than CoV-RNA1, Table 1) resulted in binding as low as 1 μM, with no free RNA remaining at an nsp7,8,12 concentration of 2.15 μM (Fig. 1h, j), thus further improved relative to an unmodified CoV-RNA2 substrate, which showed full binding at 4.4 μM (Supplementary Fig. 1b, c). The improvement of binding is not surprising, as it is well known that 2′-O-methyl groups and phosphorthioates rigidify the RNA backbone, thus enhancing their stability and protein binding^23^. Altogether, these results suggest that the 2′ oxygen of the ribose is critical for recognition by the RdRp.

### SARS-CoV-2 RdRp recognizes the O2′ group of nucleotides for elongation

**Figure 2:**
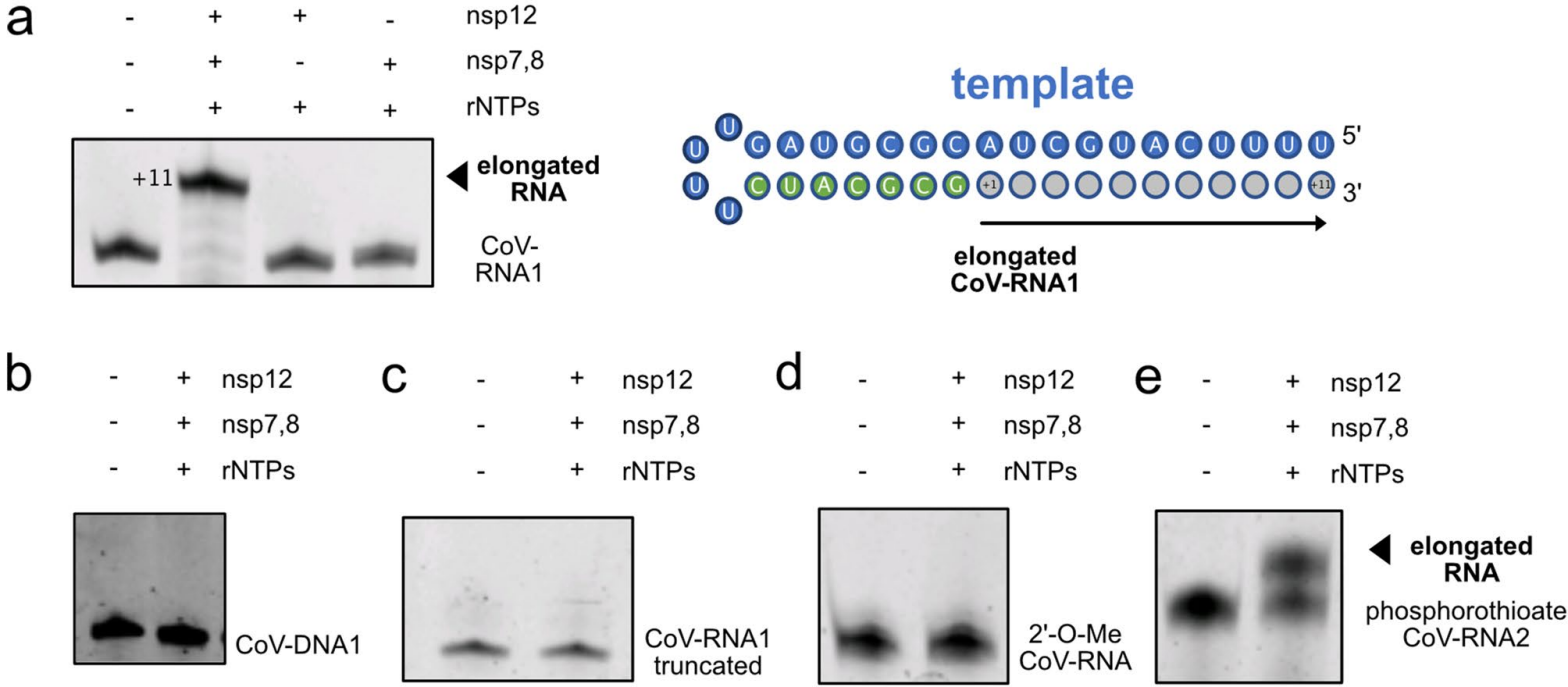
RdRp elongation activity is dependent on the functional group at the 2′ position of the ribose. (**a**) The CoV-RNA1 substrate is extended by nsp7,8,12 in the presence of rNTPs, but not by the nsp7,8 protein–protein complex, or nsp12 alone. Nsp7,8,12 does not elongate the (**b**) CoV-DNA1, (**c**) CoV-RNA1-trunctated, and (**d**) 2′O-Me CoV-RNA2 substrates, respectively. (**e**) Nsp7,8,12 inefficiently elongates the phosphorothioate CoV-RNA2 substrate in the presence of rNTPs.

We evaluated elongation activity of the aforementioned RNA and DNA substrates with the SARS-CoV-2 RdRp. In the presence of rNTPs, a fully extended CoV-RNA1 and CoV-RNA2 product was produced, as observed using denaturing polyacrylamide gel electrophoresis (PAGE) (Fig. 2a, Supplementary Fig. 1b, c). Our results also show that nsp12 alone and the nsp7,8 complex are insufficient for facilitating RNA extension and, seemingly, our RNA template does not induce nsp8 primase activity (Fig. 2a)^7^. No elongation was observed for the CoV-DNA1 substrate or the CoV-RNA1-truncated substrate, which lacks a 5′ template overhang (Fig. 2b, c). We further investigated elongation on two other RNA substrates, a fully double stranded RNA substrate (dsCoV-RNA1) and an RNA substrate containing a terminator 3′-deoxyuridine nucleotide in the 3′ terminal position of the substrate. These substrates exhibited binding with nsp7,8,12 but as expected, elongation was not observed with the dsCoV-RNA1 substrate nor for the CoV-RNA1-term substrate (Supplementary Fig. 1d–h).

Elongation assays carried out with the phosphorothioate and 2′-OMe CoV-RNA2 substrates yielded unexpected results. While the 2′-OMe CoV-RNA2 substrate bound with micromolar binding affinity, no elongation by RdRp occurred with this RNA substrate (Fig. 2d). On the other hand, elongation was observed using the phosphorothioate CoV-RNA2 substrate (Fig. 2e). Together, with the binding results, these data suggest that RNA substrate recognition by RdRp is sensitive to the 2′O position of the ribose, but elongation activity critically depends on accessibility of the 2′O of the ribose, which may be hindered by the presence of the methyl group in the 2′-OMe CoV-RNA2 substrate.

### The SARS-CoV-2 RdRp readily incorporates mismatch and 3′deoxy nucleosides

**Figure 3:**
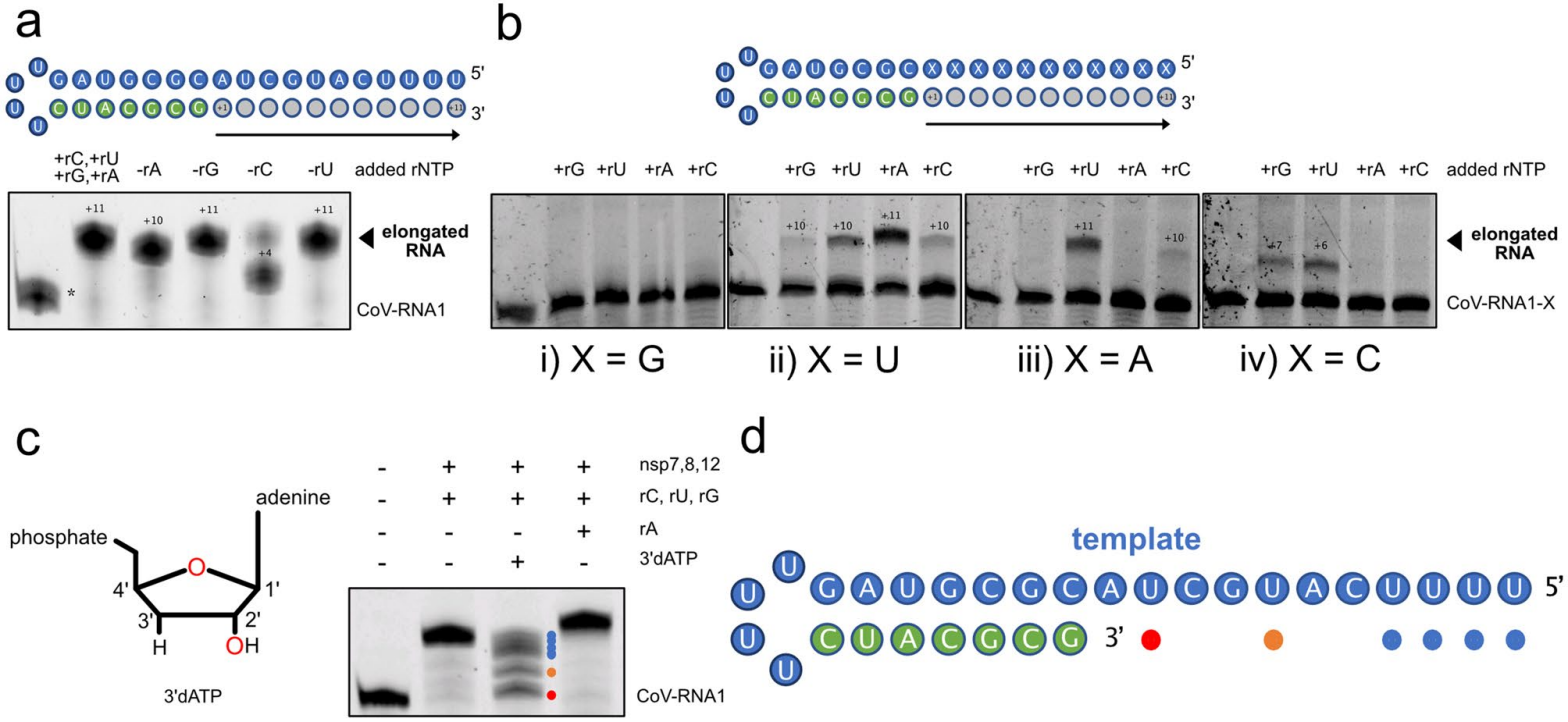
Nsp7,8,12 readily incorporates 3′dATP during extension, and lacks fidelity during extension under starved conditions. (**a**) The elongation products of CoV-RNA1 that are generated by nsp7,8,12 in the absence of a single nucleotide. The asterisk corresponds to an n + 1 RNA contaminate that was unable to be removed during substrate purifcation. (**b**) The extended products (or lack thereof) of nsp7,8,12 on uniform-nucleotide CoV-RNA1-X substrates in the presence of rNTPs. (**c**) The RdRp incorporates 3′dATP during elongation, resulting in stalled RNA products. (**c**, **d**) The secondary structure of the CoV-RNA1 substrate with positions of stalled RNA products (formed by incorporation of 3′dATP) marked by colored dots.

The SARS-CoV-2 RdRp has been shown to incorporate mismatch nucleotides, i.e. G:U pairing, during elongation^11^. We thus carried out elongation assays in the absence of one of the nucleotides in separate experiments with our CoV-RNA1 substrate to further investigate the infidelity of the RdRp (Fig. 3a, Supplementary Fig. 1i). Interestingly, when one nucleotide is left out, truncated products in the absence of rGTP, rUTP, and rATP are produced, suggesting incorporation of mismatches of all possible types by the RdRp. The least efficient elongation occurred in the absence of rCTP, with the major stalled RNA products occurring at the template G; the RdRp is inefficient at mismatching G with nucleotides other than rCTP. To further investigate this mismatch behavior, we designed four RNA substrate constructs derived from CoV-RNA1 with template overhangs comprised of only guanine, uracil, adenine, or cytosine to investigate the propensity for the nsp7,8,12 RdRp to incorporate mismatches during elongation (Fig. 3b). Interestingly, the poly-G RNA substrate resulted in no elongation when either rGTP, rUTP, rATP, or rCTP was added with nsp7,8,12 (Fig. 3b: i). This might be due to the formation of a G-quadruplex within the RNA substrate, which would sequester these nucleotides in structure, rendering them inaccessible to serve as a template for elongation by the RdRp. The poly-U, -A, and -C RNA substrates, however, were promiscuously extended by the RdRp (Fig. 3b: ii–iv): the U RNA substrate yielded extended product when the reaction was supplemented with only rATP, and to a lesser degree, rUTP, rCTP and rGTP. The A RNA substrate was extended in the presence of both rUTP and to a lesser degree, rCTP. Last, the C RNA substrate was extended in the presence of both rGTP and rUTP nucleotides. Through a process of deduction, these results, combined with elongation results carried out in the absence of one of four nucleotides, reveal the possible mismatch base pairs of the RdRp (Table 2). The RdRp mismatches A, U, and C template nucleotides during elongation, but seemingly rarely mismatches G template nucleotides. Mismatches involving uracil occur more frequently. Altogether, these results further confirm the lack of extension fidelity exhibited by the SARS-CoV-2 RdRp; in the absence of complementary nucleotides, the SARS-CoV-2 RdRp nevertheless proceeds with mismatched extension.

**Table 2:** Mismatch base pairs created by the RdRp.

RNTP omitted during elongation				
Elongation RNTP mix	no rCTP	no rATP	no rGTP	no rUTP
RNA template: COV-RNA1	At template G	At template U	At template C	At template A
Full-length product?	No	Yes	Yes	Yes
Possible base pairs	Stalls at template G	*U:C, U:G, U:U*	**[C:C]**, C:U,[**C:A]**	**[A:A]**, *A:C*, [**A:G]**
RNA template encodes for only one nucleotide				
Elongation RNTP mix	rGTP, rUTP, rATP, or rCTP	rGTP, rUTP, rATP, or rCTP	rGTP, rUTP, rATP, or rCTP	rGTP, rUTP, rATP, or rCTP
RNA template	CoV-RNA1-G	CoV-RNA1-U	CoV-RNA1-C	CoV-RNA1-A
Full-length product?	No full length	Yes	Yes	Yes
Observed base pairs	No full length	*U:C, U:G, U:U, U:A*	*C:G, C:U*	*A:U, A:C*

Bold bracket base pairs indicate mismatches that do not occur, and italic font indicates mismatches generated by the RdRp.

Possible and observed base pairs are written in the 5**′**–3**′** direction, with the template nucleotide underlined.

The majority of RNA polymerases, both human and viral, possess the ability to recognize and discriminate against modified nucleic acids, thus preventing their incorporation in the final RNA product. We carried out elongation assays with 2′-OMe and 3′dATP ribose-modified nucleotides to determine the features required or tolerated for nucleotide recognition. Similar to other polymerases (i.e. T7 polymerase^24^), the RdRp did not incorporate nucleotides modified at the 2′ hydroxyl group into an elongated product (Supplementary Fig. 1j). However, when we investigated the effect of adding Cordycepin (3′dATP), instead of rATP, to the extension reaction of our minimal RNA substrate, we observed that the SARS-CoV-2 RdRp does not discriminate against this nucleotide derivative, adding it to the RNA as if it were a natural adenosine (Fig. 3c). Furthermore, in these elongation reactions we observed six truncated RNA extension products, corresponding to the positions of the six complementary uridines in the template overhang (Fig. 3c, d). The absence of rATP, but the elongation of product beyond positions where rATP is expected to be incorporated, further support our observation on the elongation infidelity of nsp7,8,12: a U:C, U:U, or U:G mismatch is possible when rATP is omitted.

### Nsp10–nsp14 degradation relies on recognition of both the 3′OH and 2′OH groups

**Figure 4:**
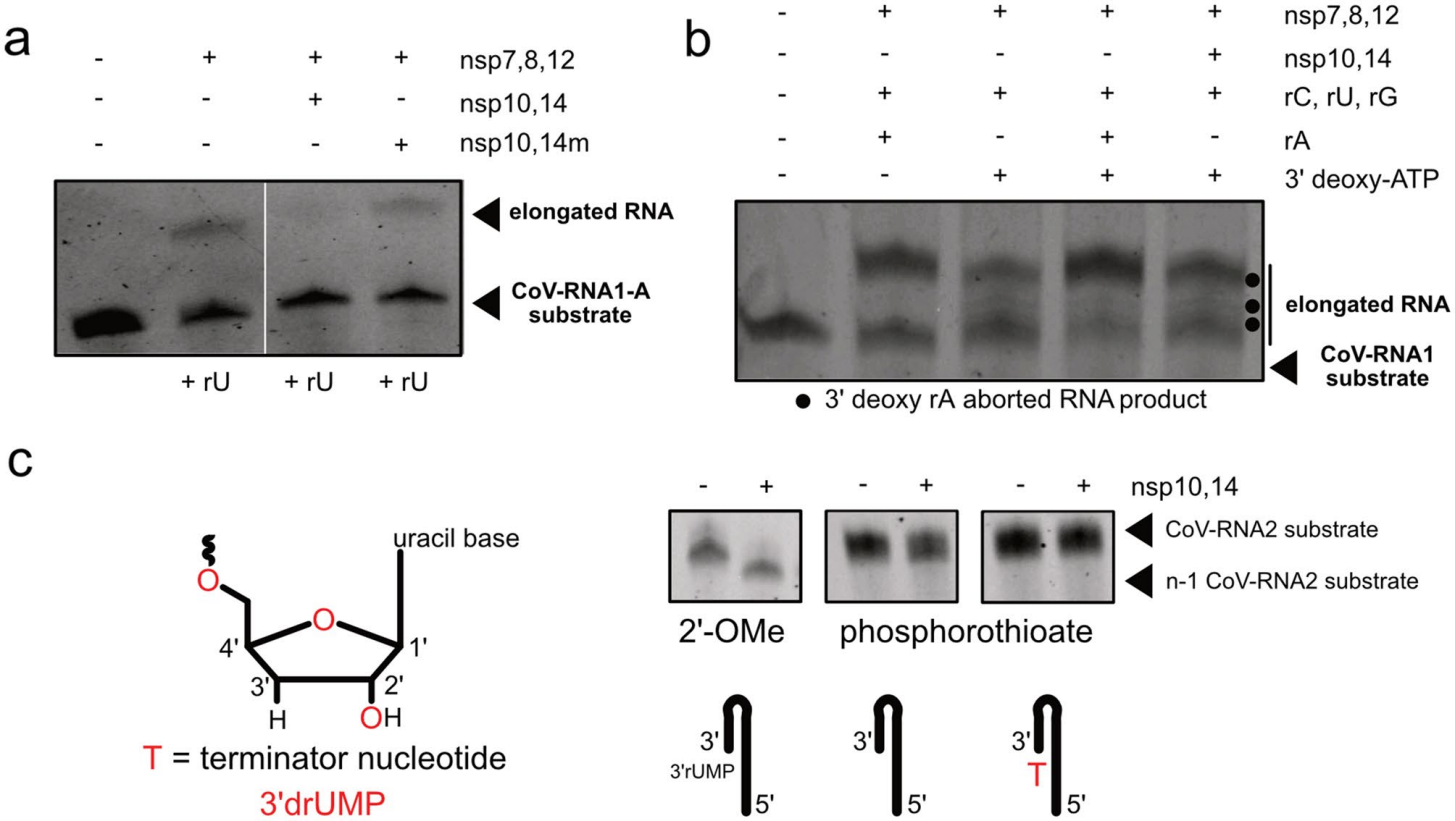
Nsp10–nsp14 degrades incorrectly matched elongated products and functions through recognition of free 3′OH groups. (**a**) Nsp10–nsp14 degrades elongated RNA products; nsp14m shows no degradation activity on elongated product. (**b**) nsp10–nsp14 is unable to degrade 3′dATP stalled RNA products produced by nsp7,8,12. (**c**) Nsp10–nsp14 exonuclease activity depends on free 2′OH and 3′OH ribose groups.

Considering the low fidelity of elongation by the RdRp, SARS-CoV genomes encode for nucleases that are reported to be responsible for the degradation of improperly elongated products^13,18,25^. Nsp14, which has been characterized as both a 3′–5′ exonuclease and more recently, an endonuclease^26^, is proposed to associate with the RdRp, and its exonuclease activity is increased by at least 35-fold when bound to nsp10^20,25^. To this end, we evaluated the nuclease activity of nsp14 in solution in the presence of nsp10 during in vitro RdRp elongation assays. Nsp14 degraded the elongated product from the CoV-RNA1-A RNA substrate (elongation was carried out with only rUTP). Mutation of critical residues for nsp14 activity (nsp14m: D90A, E92A)^27^ resulted in no significant degradation of RNA targets relative to a wildtype nsp14 control (Fig. 4a), thus confirming the role of these residues for the exonuclease activity of nsp14. It was reported for SARS-CoV-1 that nsp14 exonuclease activity is dependent on the presence of the 3′OH group of the 3′ terminal nucleotide^25^. To this end, we carried out exonuclease activity assays on elongation reactions supplemented with 3′ deoxyadenosine. Nsp14 does not degrade prematurely aborted RNAs that arise due to incorporation of this terminator nucleotide, as bands corresponding to these transcripts are still observed relative to a control that lacks nsp10–nsp14. (Fig. 4b, Supplementary Fig. 2a). These results demonstrate that nsp10–nsp14 acts as a selective proofreader. The absence of a 3′OH group on the terminal 3′ nucleotide inhibits degradation, confirming that nsp14 of SARS-CoV-2 also function as 3′ exonuclease through recognition of the 3′OH group.

It has been recently proposed that in addition to a 3′OH group, the exonuclease nsp10–nsp14 requires a 2′OH at the 3′ terminus of RNA for proofreading^28,29^. To this end we evaluated degradation activity by nsp10–nsp14 with our 2′-OMe and phosphorothioate RNA substrates (Fig. 4c). We find that addition of nsp10–nsp14 to the 2′-OMe CoV-RNA2 (in which all riboses are 2′-O-methylated, with the exception of the most 3′ nucleotide) results in a single n-1 degraded RNA product. When we add nsp10–nsp14 to phosphorothioate RNA substrates (phosphorothioate, where all nucleotides possess a phosphorothioate linkage, and phosphorothioate T, where all nucleotides possess a phosphorothioate linkage and the most 3′ nucleotide is 3′drUMP), there is no degradation of either RNA substrate. The lack of degradation of the major product further supports the notion that the presence of the 2′OH group of the ribose is required for the exonuclease activity of nsp14.

## Discussion

The fidelity of viral RNA polymerases is in general relatively low, tolerating frequent mismatches during replication^8,19,30,31^. Consequently, RNA viruses are highly variable, sometimes referred to as viral quasispecies, allowing them to easily escape therapeutic efforts and rapidly adapt to the new hosts^30,32,33^. On the other hand, such variability is associated with instability. RNA viruses usually cope with that problem by maintaining small genomes, which has its disadvantages as they may carry only limited information^34^. Some species, such as influenza viruses, carry their genetic information in small fragments^35^. Coronaviruses are amongst the largest RNA viruses, and it is essential to ensure the stability of their massive genomes. To overcome the size limits, they developed a proof-reading system based on the nsp14 exoribonuclease. RNA products generated as a result of RdRp infidelity are degraded to maintain genomic integrity.

SARS-CoV-2 shares only 79.5% sequence identity with SARS-CoV-1^36^, rendering it sufficiently divergent to be considered a new beta-coronavirus^37^. A detailed understanding of its replication activity is critical for reveal its molecular mechanisms of action, as well as for developing antiviral agents. Here, we have characterized both the RdRp (nsp7,8,12) and the role of the exonuclease nsp14 of SARS-CoV-2. The RdRp, comprised of the polymerase nsp12, nsp7 and nsp8, works concomitantly to bind RNA substrates and facilitate RNA elongation. Individually, these proteins show no detectable binding affinity or elongation of RNA, unlike other viral RNA-dependent RNA polymerases^22^. Thus, disruption of RdRp complex formation could serve as a therapeutic target against SARS-CoV-2. While extended duplexed RNA substrates are able to bind the RdRp, and only RNA substrates possessing a single-stranded 5′ template overhang allows the extension of RNA substrates.

We further show that the SARS-CoV-2 nsp12 polymerase is characterized by low fidelity, but that not all possible base pair mismatches are tolerated during elongation. RdRp is capable of incorporating U:C, U:G, U:U, C:U, and A:C mismatches when 3′ deoxy-terminator nucleotides are present. Our results show that mismatches involving G:C base pairs rarely occur, possibly associated with to the low GC content of SARS-CoV-2 genes^38^. Furthermore, our work shows that Cordycepin (3′dATP) is incorporated into elongated products indiscriminately by nsp7,8,12, and as a result, promotes the inclusion of mismatch nucleotides during elongation in vitro. Importantly, we find that recognition of a free 3′OH group is necessary for exonuclease activity by nsp14. Notably, a 2′ OH is also required as 2′-OMe oligos were not degraded by nsp14. Our results offer an alternative mechanism of action for the repurposing of Cordycepin as an antiviral against SARS-CoV-2, where it has been proposed to inhibit viral replication through an interaction with the S and M^pro^ proteins^39^. We find that the RdRp directly incorporates Cordycepin into elongated RNA products. The recognition and binding of Cordycepin by the RdRp, as recently investigated through MD simulations, further supports our experimentally-supported mechanism of action^40^. At low dosage levels, Cordycepin has been shown to be non-toxic^41–43^, and with further cellular evaluation, it could be repurposed into an antiviral drug against SARS-CoV-2. This work lays the foundation for the development of novel inhibitors that binds and block the SARS-CoV-2 replication.

Altogether, our data provide insight into the molecular mechanisms and features required by RdRp (nsp7,8,12) to recognizes and elongate RNA substrates. It sheds new light to the unique roles of the nsp14 exonuclease and nsp10. Finally, our findings suggests that development of novel therapeutic approaches for treatment of SARS-CoV-2 can be based on RNA-derived oligonucleotides with 3′ deoxy-terminator nucleotides to inhibit replication, combined with modifying 2′OH and 3′ OH recognition.

## Material and methods

### RNA preparation

DNA template containing the T7 promoter region for the RNAs shown in Supplementary Fig. 1 were ordered from IDT and transcribed in vitro using in-house prepared T7 polymerase. Briefly, DNAs were diluted to 8 μM and supplemented with 8 μM T7 promoter primer (Eurofins Genomics), 20X transcription buffer (100 mM Tris pH 8, 100 mM spermidine, 200 mM DTT), 5% PEG 8000, 8 mM of each rATP, rUTP, rGTP, and rCTP, 40 mM MgCl_2_, and 0.03 mg of T7 polymerase. Transcription reactions were run at 37 °C for 1 h, followed by denaturing purification on a 20% urea polyacrylamide gel, run at 10 W for 1 h. The RNA was excised from the gel and extracted using the crush and soak method^44^. The extracted RNA was equilibrated against 6 volumes of water and then concentrated to 1 mg/mL. All other RNAs used in this study were purchased from IDT as PAGE purified and desalted oligos.

### Elongation and binding shift assays

For both elongation and binding shift assays, RNAs were diluted to 20 ng/μL in buffer containing 10 mM Na-HEPES pH 7.4 and 50 mM NaCl, and then snap cooled (heated to 95 °C for 5 min, followed by immediate cooling on ice for 5 min) prior to incubation with nsp proteins. For nsp7,8,12 complex assembly, 20 μM nsp12 was incubated for 10 min with 60 μM nsp7 on ice, followed by the addition of 60 μM nsp8. The complex was incubated for 30 min on ice and further purified using size exclusion chromatography (to ensure complex formation. Supplementary Fig. 2c) before being added to the RNA substrates. For extension assays, 5 μL of 20 ng/μL RNA was added to 5 μL of elongation buffer (20 mM Na-HEPES pH 7.5, 100 mM NaCl, 5% v/v glycerol, 10 mM MgCl_2_ and 5 mM β-mercaptoethanol), and 5 μL of nsp7,8,12 pre-formed complex (final concentration of 6.6 μM nsp12, 20 μM nsp7 and 20 μM nsp8). The RNA protein mix was incubated at room temperature for 5 min before the addition of 1 mM (final concentration) of each rNTP. Cordycepin was purchased from Sigma Aldrich. Reactions were carried out at 37 °C for up to 30 min and quenched with the addition of 50 mM EDTA and 1,000 units of proteinase K, followed by 5 additional min of incubation at 37 °C. 6X denaturing buffer (8 M urea, 10 mM Tris pH 8.1, 40 mM EDTA) was then added to the reactions, followed by incubation at 95 °C for 5 min and subsequent loading onto a denaturing 20% urea polyacrylamide gel. Gels were run at 10 W in 1X TBE for 1 h, followed by SYBR-Gold staining and imaging using GelDoku Biorad software. Gels were cropped to show relevant regions in the main figures; full gels are shown in Supplementary Figs. 3 and 4. For binding assays, increasing amounts of protein (as indicated in the figures) was added to RNA substrate (diluted in the elongation buffer) and supplemented with 4% v/v glycerol (final concentration). Reactions were incubated at room temperature for 5 min prior to loading on a nondenaturing 20% polyacrylamide gel. Gels were run under the same conditions as described for extension assays. All elongation and binding shift assays were carried out with at least two biological replicates.

### Degradation assays

Degradation assays were carried out by supplementing the elongation assay (as described above) with nsp10–nsp14 at equimolar concentrations to nsp7,8,12. For RNA only substrate degradation assays, the optimal nsp10–nsp14 concentration and degradation time for non-elongated RNA substrates was determined in control degradation assay experiments, as depicted in Supplementary Fig. 2b.

### Protein expression and purification

Nsp12 and nsp8 were expressed as previously published^5^. The gene encoding the nsp7 sequence (SARS-CoV-2 nsp7, 1–83) was ordered from Integrated DNA Technologies (IDT-Coralville, USA) optimized for *E.coli* expression and cloned using the Slice method^45^ into the petM5a vector (optimized petM11^46^) . The constructs of nsp10, comprising the amino acids 4254–4392 and nsp14, comprising the amino acids 5926–6452, of SARS-CoV-2 polyprotein 1ab were optimized for expression in E. coli and ordered from GeneArt; constructs were then subcloned into pETDUet-1 expression plasmid using BamHI and NotI restriction enzymes. Nsp7 plasmid was transformed into *E. coli* BL21 (DE3) and nsp10 and nsp14 plasmids were transformed into Rosetta (DE3) pLysS competent cells, separately for single expressions and co-transformed to generate the complex of both proteins. Transformed cells were cultured at 37 °C in TB media containing 100 mg/L kanamycin or 100 mg/mL ampicillin for nsp7 and nsp10 or nsp14, respectively. After the OD_600_ reached 2, the culture was cooled to 18 °C and supplemented with 0.5 mM IPTG. After overnight induction, the cells were harvested through centrifugation, and the pellets were resuspended in lysis buffer (20 mM Tris-HCl, pH 8.0, 150 mM NaCl, 4 mM MgCl_2_, 10% glycerol, 10 mM imidazole, 0.1% triton X-100, 10 μg/mL DNAse I and protease inhibitor cocktail) and lysed by sonication. The insoluble material was removed through centrifugation. The fusion protein was first purified by Ni–NTA affinity chromatography. Supernatant was applied to nickel column, 5 mL, and washed extensively with lysis buffer, followed by then 20 mL of buffer supplemented with 20 mM imidazole and eluted with 20 mM Tris-HCl, pH 8.0, 150 mM NaCl, 4 mM MgCl_2_, 10% glycerol, 350 mM imidazole. The N-terminal 6-histidine tag was removed by TEV protease and the solution was dialyzed against a buffer containing 20 mM Tris-HCl, pH 8.0, 150 mM NaCl, 4 mM MgCl_2_, 10% glycerol, 10 mM imidazole and 1 mM β-mercaptoethanol. After 16 h, the protein was purified over a nickel column, the flow though was collected (containing the cleaved nsp7 or nsp10 or nsp14) concentrated and applied to a size exclusion chromatography column (S200) pre-equilibrated with 20 mM HEPES pH 7.5, 150 mM NaCl, 5% glycerol, 1 mM TECEP 1 mM MgCl_2_ for nsp7, nsp10 and nsp14, or with 50 mM Tris-HCl, pH 8.0, 150 mM NaCl, 5 mM MgCl_2_, 2 mM β-mercaptoethanol, for the nsp10–nsp14 complex. To further remove possible impurities from nsp10–14 complex, the sample was diluted to 50 mM NaCl with 50 mM Tris-HCl, pH 8.0, 5 mM MgCl_2_, 2 mM β-mercaptoethanol, concentrated and applied to an ion-exchange chromatography column (Q FF, 1 mL) equilibrated with 50 mM Tris-HCl, pH 8.0, 50 mM NaCl, 5 mM MgCl_2_, 2 mM β-mercaptoethanol. The purified protein elutes first, where the impurities are bound to the resin and eluted by sequentially increasing the NaCl concentration. For all the proteins, its purity was assessed by SDS gel (Supplementary Fig. 2c, d), concentrated to 2 mg/mL, flash frozen in liquid N2 and stored at − 80 °C until further usage.

## Supplementary Information

Supplementary Figures.
