# Supplementary material for: Characterization of SARS-CoV-2 replication complex elongation and proofreading activity
Source: Sci Rep. 2022 Jun 10;12:9593. doi: 10.1038/s41598-022-13380-1 (PMC9185715; doi:10.1038/s41598-022-13380-1)
Supplement: Supplementary file 1 — Supplementary Figures. [file 41598_2022_13380_MOESM1_ESM.docx]

**Supporting information: Characterization of SARS-CoV-2 elongation and proofreading activity**

Alisha N. Jones^1,2^, André Mourão^1,2^, Anna Czarna^3^, Alex Matsuda^3^, Roberto Fino^1,2^, Krzysztof Pyrc^3*^, Michael Sattler^1,2*^, Grzegorz M. Popowicz^1,2*^

^1^ Institute of Structural Biology, Helmholtz Zentrum München Ingolstädter Landstr. 1, 85764 Neuherberg, Germany.

^2^ Biomolecular NMR and Center for Integrated Protein Science Munich at Department Chemie, Technical University of Munich, Lichtenbergstraße 4, 85747, Garching, Germany.

^3^ Virogenetics Laboratory of Virology, Malopolska Centre of Biotechnology, Jagiellonian University, Gronostajowa 7a, 30-387 Krakow, Poland.

^*^To whom correspondence should be addressed. Tel: +49 (0)89 3187 3727; Email: [Grzegorz.popowicz@helmholtz-muenchen.de](mailto:Grzegorz.popowicz@helmholtz-muenchen.de). Correspondence may also be addressed to [sattler@helmholtz-muenchen.de](mailto:sattler@helmholtz-muenchen.de) and k.a.pyrc@uj.edu.pl

**Supplementary Figures**

**Supplementary Figure 1**

Supplementary Figure 1. a, Nondenaturing gels showing that nsp 12 alone, and the nsp 7,8 protein-protein complex do not strongly bind RNAs lacking a template overhang. The stem-loop sequence is shown on the left, with the apical loop sequence represented by XXXX which is shown above their corresponding lanes in the gels. b, The secondary structure of an RNA substrate recognized by the SARS-CoV-2 RdRp, coined CoV-RNA2 and elongation of CoV-RNA2 by the RdRp. c, a nondenaturing gel showing binding of nsp 12,7,8 with CoV-RNA2. d, The secondary structure of dsCoV-RNA1 substrate. e, a nondenaturing gel showing binding of nsp 12,7,8 with dsCoV-RNA1 f, The secondary structure of a RNA substrate possessing a 3’ terminal deoxyuracil nucleotide, coined CoV-RNA1-term. g, The CoV-RNA1-term substrate binds with nsp 12,7,8 between 2.4 and 4.4 μM. h, No elongation occurs with the CoV-RNA1-term substrate due to the lack of a 3’OH group. The asterisk corresponds to an elongated product due to slightly impure starting template. i, Replicate gel (Figure 3a): nsp7,8,12 lacks fidelity under starved conditions. j, Elongation reaction with 2’-OMe modified nucleotides.

**Supplementary Figure 2**


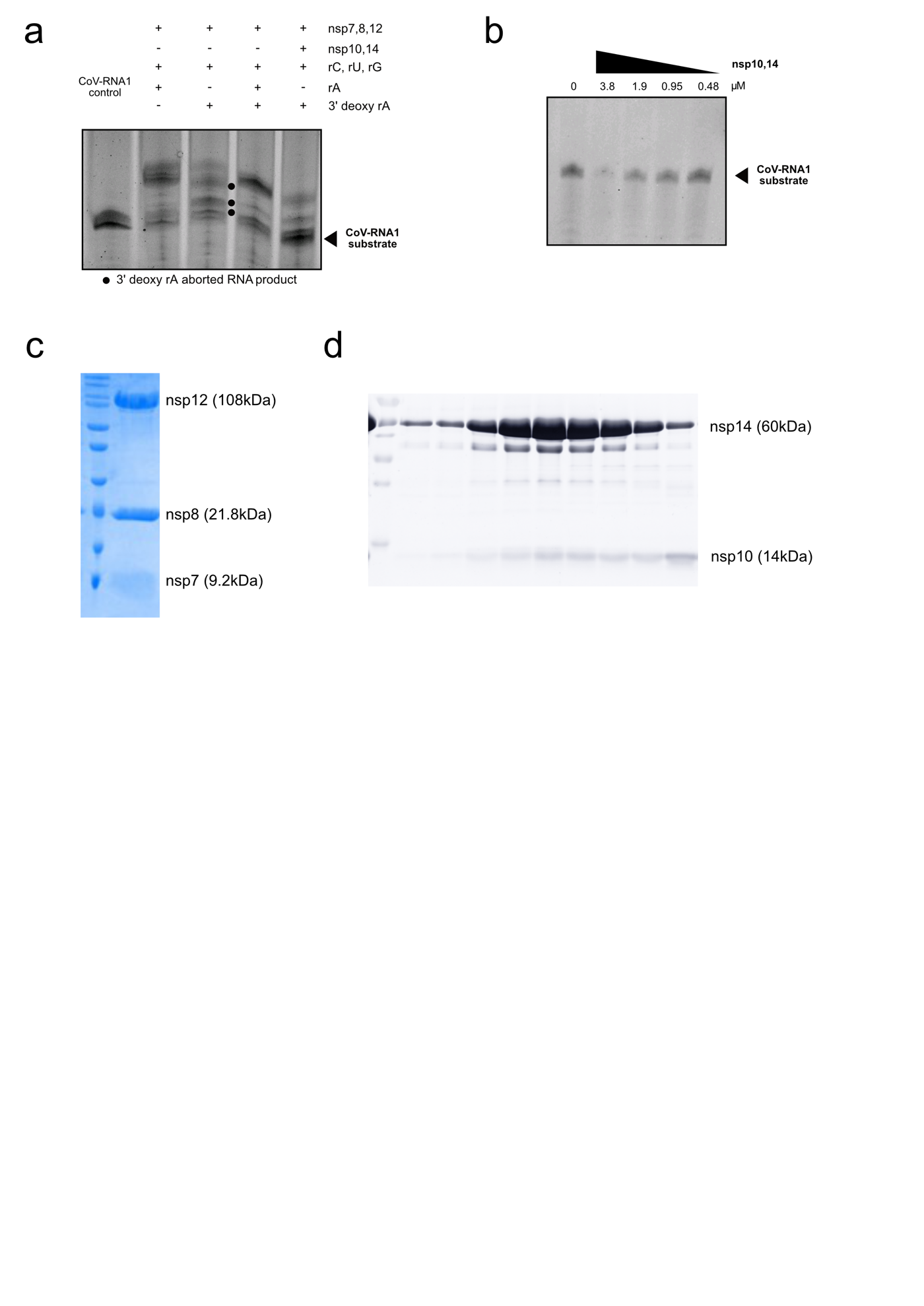


Supplementary Figure 2. a, Replicate gel (Figure 4b): nsp10-nsp14 is unable to degrade 3’drATP stalled RNA products produced by nsp7,8,12. b, Concentration optimization for assessing nsp10,14 exonuclease activity. b, Coeluted SEC product of the nsp7,8,12 RdRp as evaluated by SDS gel analysis. c, Coeluted SEC product of the nsp10,14 exonuclease complex, as evaluated by SDS gel analysis. Slight degradation of nsp14 is apparent with time.

**Supplementary Figure 3**


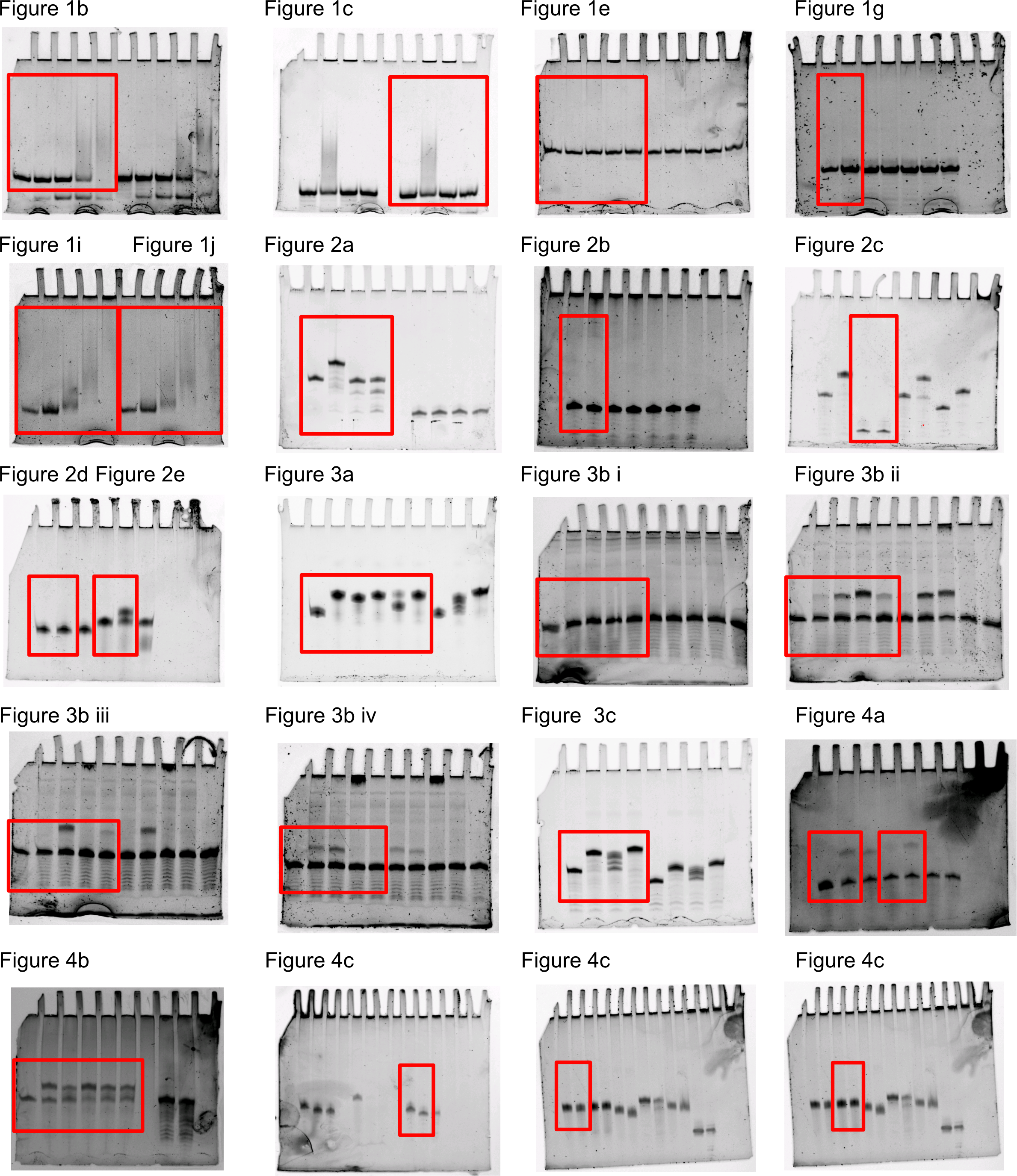


Supplementary Figure 3. Uncropped gels corresponding to all gels shown in Main Figures 1-4. Red boxes indicate the cropped region of the gel shown in the Main Figures.

**Supplementary Figure 4**


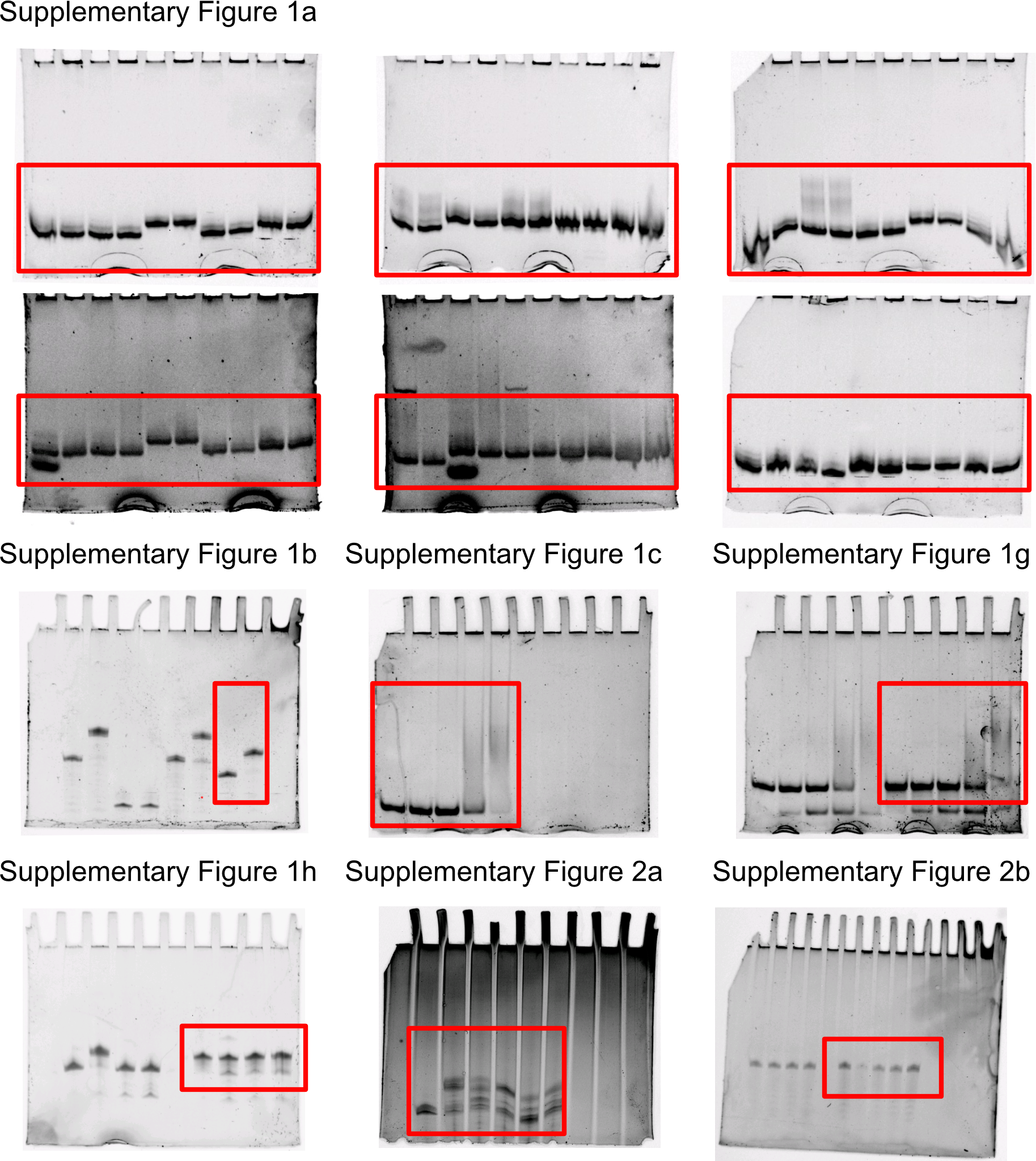


Supplementary Figure 4. Uncropped gels corresponding to gels shown in Supplementary Figures 1 and 2. Red boxes indicate the cropped regions observed in the Supplementary Figures.
